# Optimising Investment in Health Innovations in Europe

**DOI:** 10.3390/jmahp14010011

**Published:** 2026-02-13

**Authors:** Tosin Adeyemo, Tim Wilsdon, Christina Vandorou, Fiona Davies, Annabelle Godet

**Affiliations:** 1 Charles River Associates, London EC2M 7EA, UK; twilsdon@crai.com; 2 Johnson & Johnson Innovative Medicine, High Wycombe HP12 4EG, UK; 3 Johnson & Johnson Innovative Medicine, 92787 Issy-Les-Moulineaux, France

**Keywords:** health, pharmaceuticals, expenditure, investment, value, cost containment

## Abstract

European citizens made it clear in 2024 that healthcare should be the EU’s top priority for shaping the future of Europe. This sentiment reflects the escalating health challenges facing the region, driven by ageing populations, rising chronic disease burdens, and persistent disparities in access to healthcare. Despite these growing needs, the most recent data on health spending as a share of gross domestic product (GDP) is just slightly above the pre-COVID-19 pandemic level, and spending on pharmaceuticals specifically has remained a stable proportion of healthcare spending over the last 20 years. Austerity measures have profoundly impacted the health sector and pharmaceutical industry, more so than any other sectors, despite the wide range of health and socioeconomic benefits medicines bring to patients, the health system, and society. Such trends are not keeping pace with evolving population demographics and disease prevalence. To secure a healthier, more equitable future, Europe must urgently increase health investments, optimise health systems, and address unmet needs by supporting fast uptake of pharmaceutical innovations. Policymakers must work with all stakeholders to ensure stronger and sustained investments in health innovations by (i) adopting a long-term vision, moving away from short-term thinking and valuing health appropriately to drive economic growth; (ii) implementing transformative policies that eliminate ineffective and wasteful spending; (iii) promoting value-based approaches to improve patient access and system sustainability, and (iv) creating incentives that attract greater investments to strengthen Europe’s competitiveness and safeguard against health threats.

## 1. Introduction

Following the 2008 financial crisis, European Union (EU) member states implemented austerity measures to reduce government debt and spending [1]. Many EU Member States implemented cuts to public expenditure, cancelled planned public investment and imposed financial constraints, including reductions in public health funding. The hospital sectors and pharmaceuticals (namely innovative medicines) were among the areas most significantly affected by these cuts [2]. With constrained budgets, European governments imposed restrictions that delayed access to new innovative medicines, limited their prices, and restricted their use to certain populations. Clawbacks were also introduced to control the growth in spending and compensate for any ‘overspend’. These policies have only accumulated over the last 15 years.

Today, we are at a crossroads. Five years on, still in the wake of the COVID-19 pandemic and sluggish growth, now with high inflation and mounting geopolitical pressures, austerity continues to drive health policy. We have already seen significant changes through country-specific policies focused on cost, including new reimbursement rules and updated clawback mechanisms [3]. As highlighted by the Draghi Report in 2024 Europe’s competitiveness is in the global spotlight [4]. The region faces growing pressure to increase its spending on innovation and to pay a fair share of global investment in the research and development (R&D) of innovative medicines. Addressing this spending challenge is critical to Europe’s competitiveness.

We acknowledge that European governments must manage constrained budgets amid sluggish economic growth while balancing the competing objectives of fostering innovation and ensuring affordable access. However, the question remains whether the savings achieved through short-term pharmaceutical cost containment outweigh the negative impacts on health outcomes, innovation, and economic growth and are therefore warranted. We believe that finding a sustainable balance between affordability today and funding future medical breakthroughs is a difficult yet essential challenge for strengthening competitiveness and protecting Europe’s future.

In this article, we examine issues related to (i) trends in Europe’s health spending relative to population needs; (ii) value of investing in health and pharmaceutical innovation; (iii) trends in pharmaceutical cost containment in Europe; (iv) the impact of cost containment and (v) policy considerations for delivering optimal healthcare in a financially constrained environment.

## 2. Trends in Europe’s Health Spending Relative to Population Needs

In 2024, European citizens made it clear: healthcare should be the EU’s top priority for shaping the future of Europe [5]. This sentiment reflects the escalating health challenges facing the region, driven by ageing populations, rising burden of chronic diseases, and persistent disparities in access to healthcare.

The health challenges facing Europe are significant. In 2022, over 1 million premature deaths (under age 75) occurred in the EU, with 64% preventable through public health measures and 36% treatable with timely healthcare interventions [6]. Non-communicable diseases (NCDs), including cardiovascular diseases, cancer, diabetes, and respiratory conditions, account for 90% of deaths and 80% of the health burden in the EU [6]. The prevalence of chronic conditions is particularly stark among older adults, with four out of five individuals aged 50 and above living with at least one chronic condition, and half managing multiple conditions [7]. Cancer cases are projected to increase by a staggering 77% globally by 2050, posing a major threat to European health systems [8]. Mental health disorders also demand attention, with nearly half the EU population—46%—currently experiencing mental health-related problems [9]. The prevalence of depressive disorders in the World Health Organisation (WHO) European Region increased from 4.6% in 2019 to 5.2% in 2021, while the prevalence of dementia is expected to double by 2030 [10].

These challenges are intensified by Europe’s ageing population. The old-age dependency ratio is rising as the proportion of elderly citizens grows relative to the working-age population, driving increased demand for healthcare and long-term care services [11]. This demographic shift places unprecedented pressure on health systems already grappling with workforce shortages and resource constraints [11].

The average health expenditure as a share of GDP saw a growth during the COVID-19 pandemic in 2020/2021 but has since declined, remaining slightly above the pre-pandemic level, though with wide variation across European countries [12]. Spending on pharmaceuticals specifically has remained a stable proportion of healthcare spending over the last 20 years [13]. These trends are evident despite significant unmet needs, increased use of medicines [14], and numerous groundbreaking therapies launched during the same period. The evidence highlights an alarming disconnect between population health needs and resource allocation in Europe. In our view, this disconnect not only impedes efforts to address today’s health needs but also reduces investment to develop tomorrow’s cures.

## 3. The Economic Imperative of Investing in Health

Healthcare is not a cost to be minimised but a long-term investment in Europe’s future. Indeed, investing in health today builds a healthier, more productive tomorrow. A positive feedback loop exists whereby greater investment in healthcare drives better health outcomes, and a healthy population maximises productivity, which in turn generates economic growth in terms of increase in GDP per capita [15]. The economic case for prioritising health is compelling: every €1 invested in health results in direct improvements in population health and yields more than double the return in economic benefits [16].

McKinsey (2021) estimates that these benefits could total €2.4 trillion across Europe by 2040, attributed to increased labour supply, higher productivity, and reduced healthcare costs [16]. Mounting evidence documents the economic power of healthy populations. For example, a 10% increase in adult survival rates was shown to correlate with an 11% boost in labour productivity [17], and, in high-income countries, each one-year gain in life expectancy can bring benefits equivalent to 4–5% of GDP (based on the Value of a Statistical Life) [18]. This illustrates the importance of moving away from siloed thinking in health policy and taking a broader lens. Returns may not be realised within the same sector, yet these positive spillovers remain vital to the economy.

In reality, it is poor health that comes at a significant cost to society. For instance, poor health costs Europe, on average, about €2.3 trillion (15% of GDP annually) in lost economic opportunity, equivalent to about €4350 per person [16]. In 2018, four major NCDs alone, including cancers and mental health disorders, resulted in a 2% loss in GDP across the EU [7]. In the same year, direct costs from cancers were €97 billion across the EU, while indirect costs were €66 billion in productivity losses and €25 billion in informal care costs [19]. Delay in patient access to some cancer medicines may have led to a potential loss of more than 30,000 life years in Europe [20]. For mental health disorders, an analysis of their economic impact reveals that, in 2018, poor mental health in the EU cost over €600 billion, including €190 billion in healthcare, €170 billion in social security, and €240 billion in lost income [21]. The economic value of life years lost due to mental health disorders could be up to nearly 8% of GDP [22].

With ageing populations, rising chronic disease burdens, and fiscal constraints, Europe must view health investments—spanning prevention, early diagnosis, and innovative treatments—as critical drivers of economic growth and societal resilience.

### 3.1. Disease Prevention

Investing in disease prevention efforts such as vaccinations and preventative treatments have been shown to yield substantial health and economic returns by reducing healthcare costs, increasing workforce participation, and boosting productivity.

- Disease prevention yields high returns on investments. Research shows that 85% of potential health gains stem from preventing ill health associated with chronic diseases [16]. Every €1 invested in national public health prevention in high-income countries yields approximately €27 in benefits, while investments in protective interventions, such as vaccinations, yield about €34 [23]. In mental health, every €1 invested in interventions to prevent and treat adolescent mental disorders returns €24 in benefits [24]. Investing in disease prevention also drives economic growth. For example, in the UK, a 20% reduction in the incidence of 6 major disease categories, mainly NCDs, could raise GDP by an estimated 0.7% within 5 years—an annual boost of £19.8 billion [25].
- Vaccinations save lives, reduce disease burden and contribute to cost savings in healthcare. Globally, 4–5 million deaths are prevented each year due to vaccinations [26]. Adult vaccines can return up to 19 times their initial investment in benefits for population health, healthcare systems, and society [27]. For every €1 spent on childhood immunisations, nearly €11 is saved in societal costs [28]. Investing more to prevent vaccine-preventable cancers, such as human papillomavirus and hepatitis B, could have avoided up to €18 billion in productivity losses across Europe [29].

### 3.2. Early and Accurate Disease Diagnosis

Achieving early and accurate diagnoses can improve the patients’ survival prospects, health outcomes, and reduce healthcare costs [30].

- Screening enables early diagnosis and reduces mortality. In the UK, there was a 25% reduction in mortality (after 10 years of follow-up) in breast cancer patients diagnosed from screening [31]. Evidence shows an 18% reduction in mortality in lung cancer in Europe due to screening every 3 years, together with smoking cessation programmes [32].
- Early diagnosis of cancer averts significant treatment costs. Diagnosing cancer patients early not only ensures better survival rates, fewer complications, sustained quality of life and supports the financial sustainability of healthcare systems [33]. Indeed, treatment for patients diagnosed with cancer early has been shown to be 2–4 times less expensive than that for those diagnosed with more advanced disease [34].
- Next generation sequencing enables accurate diagnosis, improves treatment accuracy and reduces unnecessary healthcare costs. In France, an investment of less than €2 million in a genetic test to detect EGFR mutations in lung cancer patients to guide targeted therapy saved the health system a total of €459 million by testing for the EGFR biomarker [35]. Similarly, in Ireland, a study has shown that an investment of about €24,000 in pre-screening for genetic side effects in fluoropyrimidine chemotherapy saved the health system approximately 10 times the initial cost (€232,000) in hospitalisation expenses [36].

### 3.3. Treatment with Innovative Medicines

A plethora of evidence documents correlation between the use of innovative treatments and improvements in health outcomes, healthcare cost savings, and population productivity, leading to a range of economic benefits. In fact, research has shown that 73% of the observed increase in life expectancy in high-income countries between 2006–2016 was due to new pharmaceutical treatments [37].

- Use of innovative treatments improves health outcomes, reduces preventable deaths and extends healthy lives [37]. In Europe, we have seen continuous improvements in 5-year survival rates for the most common cancer types—driven by multiple factors, including a step change in the availability of innovative treatments over the past 20 years [38,39]. Today, an HIV-positive adult receiving antiretroviral therapy can have a life expectancy approaching that of the general population [40]. Cure rates among hepatitis C (HCV) patients have increased from 41% to 96% within 16 years due to improved treatments [41].
- Innovative treatments can deliver significant savings in healthcare costs. Studies indicate that long-term estimated cost savings from launching 17 new drugs in Europe amount to €58 billion [42]. The use of monoclonal antibodies in the management of asthma has been found to significantly reduce exacerbations. It is associated with improvements in asthma symptoms and quality-of-life scores, and reductions in medication use, health resource utilisation, and hospital visits [43]. In Ireland, these led to cost savings of €894 per patient over a 6-month period [44,45].
- Innovative medicines can improve population productivity by enabling patients to return to work. For example, melanoma therapies in the EU27 increased productivity by 3.8 million hours and labour income by €391 million between 2011 and 2022 [46]. In Germany, each novel treatment launched was estimated to have saved approximately 200 working years per year during the observation period (1988–2004) [47].

## 4. Trends in Pharmaceutical Cost Containment in Europe

**Figure 1:**
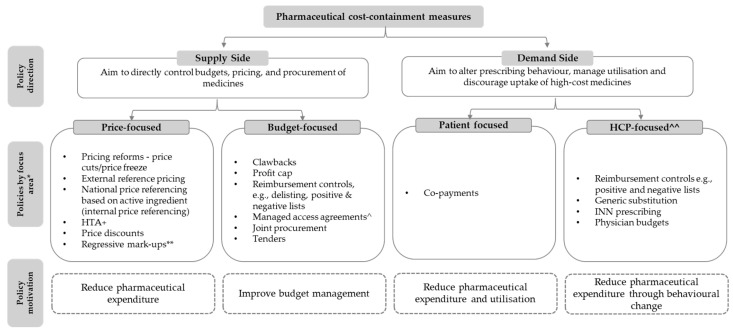
A taxonomy of pharmaceutical cost containment in Europe. * Policies are grouped into focus areas based on the primary intent of the policy; focus areas are not mutually exclusive. ** Mainly affects downstream sector (wholesalers and retailers). ^ Combination of a budget control measure and data collection agreement [52]. ^^ Physician or pharmacist facilitated. + HTA (health technology assessment) can be relevant to both price and (sometimes) budget.

Several studies have examined the different types of measures used by governments to contain healthcare and pharmaceutical costs. Stadhouders et al. (2016) identified 2250 cost-containment policies, including price controls, volume controls, market-oriented cost-containment measures, and budgeting, implemented across all 34 OECD member states from 1970 to 2015 [48]. Other studies, including Donato et al. (2022) [49], Nguyen et al. (2015) [50], and Carone et al. (2012) [51] specifically examined pharmaceutical cost-containment measures in certain countries, and such measures can be generally classified into two categories: supply side and demand-side policies (Figure 1).

Evolution in pharmaceutical cost-containment measures across Europe can be considered according to the policy motivation, that is, the aim of undertaking such action, and from the type or format of measure enforced.

### 4.1. Evolution in Policy Motivations for Cost Containment

Policy motivations for cost containment in Europe seem to differ across the two periods. Following the 2008 financial crisis, the focus of policymakers was primarily based on fiscal considerations. Austerity measures were introduced to reduce government spending in the healthcare sector and control deficits [51,53,54,55]. The challenges facing policymakers in 2025 are even more complex and multifaceted, combining continued fiscal pressure post-pandemic, with the need to ensure health and social care for a more demanding population, as well as concerns for managing industrial production and competitiveness, supply resilience and geopolitical pressures) [3,4]. There is still a focus on prioritising short-term savings, viewing healthcare as a cost instead of an investment [56,57].

### 4.2. Evolution in Cost Containment Measures

There is a body of literature on cost-containment measures implemented during and in the immediate aftermath of the 2008 financial crisis. There is also recent literature on different types of cost-containment measures being implemented today. However, no literature has specifically evaluated the evolution of measures over the two periods. For example, several studies, including Carone et al. (2012), have comprehensively reviewed pharmaceutical cost-containment measures in the wake of the financial crisis across Europe [51,53,54,55]. These studies show that between 2010 and 2012, a significant number of cost-containment measures were introduced across 23 EU countries, including discounts, clawbacks and rebates; price freezes and cuts; changes in value-added tax; changes in external reference pricing; changes in internal reference pricing; and stringent reimbursement criteria [51,53]. Additionally, several European countries restricted access by increasing user charges and raising co-payments [54,55]. More than a decade after the financial crisis, cost-containment measures are intensifying and are further exacerbated by recent geopolitical pressures. We can explore how these measures have evolved according to four major themes.

- Pricing restrictions are increasingly being used as a form of cost containment. For example, in Germany, the German Stabilisation Act, enacted in 2022, introduced a de facto reduction in the period of free pricing from 12 to 6 months to further contain costs as well as “guardrails” (i.e., pricing caps) into the process to curb rising pharmaceutical expenditures [58]. In Denmark and France, price cuts remain a key measure for containing pharmaceutical costs [59,60].
- Budget-control measures such as clawbacks are increasingly being used across Europe. There has been a marked increase in the impact of clawbacks on pharmaceutical sales in Europe over the last decade. A recent analysis of clawbacks in Europe, based on data on 24 medicines across six countries (France, Germany, Italy, Spain, Switzerland, and the United Kingdom), found that the growth rate of clawbacks (in absolute terms) currently exceeds the increase in sales by roughly 20% per year. If this continued, clawbacks would exceed 100% of all cohort’s sales by 2033 [61]. In France, clawback levels increased more than 20-fold over a 3-year period, from €0.1 billion in 2019 to more than €1.6 billion in 2023 [57]. In Greece, the value of clawbacks and rebates increased more than 4-fold between 2012 and 2019 [62]. A similar trend was observed in the UK, where clawback levels have increased 5-fold over 2 years (from £0.6 billion in 2021 to £3.3 billion in 2023) [63]. These unsustainable levels of clawbacks are unparalleled compared to other sectors.
- Restrictive reimbursement criteria are impacting the breadth of patient access. Regulatory agencies have evolved to enable timely authorisation of promising new health technologies, which may not align with the extensive evidence requirements for HTA. Combining this with budget-impact considerations being increasingly influential [64] can result in more restricted access to innovation in many countries, for example, by limiting the reimbursed patient population. An analysis of HTA outcomes in France, Germany, the Netherlands, and Sweden showed a decrease in the number of positive HTA recommendations in 2023 compared to the average between 2019 and 2022 [65]. In France, increasing stringency of its early access scheme (AAP) criteria has led to a decline in approval rates (for pre-marketing authorization) from 85% in 2021 to 49% in 2024 [66]. With the increasing stringency of reimbursement criteria, the use of managed entry agreements has grown at an average annual rate of 24% [67]. It is vital that MEAs are not misused in ways that unjustifiably restrict or delay patient access. While the recently enacted EU HTA regulation may help streamline clinical assessments across the region by avoiding duplication, decisions on pricing and reimbursement remain the prerogative of individual Member States [68].
- Policies to promote the use of generics and biosimilars are gaining prominence. In Denmark, the Medicine Council announced that it would no longer assess biosimilar medicines, and that they would be automatically included in tenders [69]. Automatic substitution policies for biosimilars are being implemented in at least five countries, including Czechia, Estonia, France, Latvia, and Poland [70].

**Figure 2:**
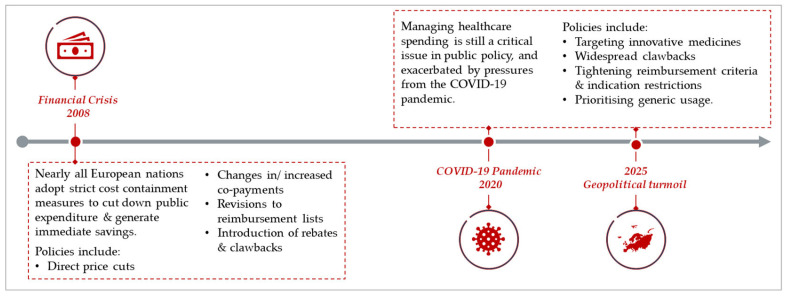
A timeline of the evolution of cost containment policies in Europe.

Some key differences between pharmaceutical cost-containment measures implemented during the financial crisis and those implemented in the post-financial crisis period are apparent (Figure 2).

- Post-financial crisis period, nearly all European nations adopted strict cost-containment measures to cut down public expenditure and generate immediate savings. The most common policy changes applied were price cuts, changes in copayments, updates to reimbursement lists, and rebates/clawbacks. Countries most affected by the crisis (e.g., Greece, Portugal, Baltic countries) implemented the most policy changes [53,54].
- Today, managing healthcare spending is still a critical issue in public policy and healthcare reform, especially with increasing budgetary pressures. More targeted approaches focus on higher-cost innovative medicines now compared to prior periods of austerity, but politically challenging policies, such as co-payments, are less likely to be introduced. Tightening of reimbursement criteria continues thus making early access approaches increasingly important for patients. Finally, cost-containment policies are more likely to be included in a holistic package of measures than ad hoc rule changes.

## 5. Impact of Cost Containment Measures: Lessons from the Financial Crisis Period

While there may be differences in the forms of pharmaceutical cost-containment measures used today compared with those during the financial crisis, it appears that the lessons learned from their impact are not being considered by policymakers. The negative effects of healthcare austerity measures on patients, health systems, and society across Europe are well-documented [71]. Specifically, cost containment in healthcare:

- Does not address system inefficiencies. It is estimated that 20% of all health spending in Europe is waste [72]. Yet, there is no evidence that cost containment measures reduce waste and inefficiencies in health systems [73,74]. For example, in Italy, an analysis of the impact of the cost containment measures implemented between 2010 and 2013 (after the financial crisis) on efficiency and productivity of public hospitals shows that the cost containment measures led to a reduction in both spending and outputs (such as hospital discharges), but did not improve nor reduce hospital efficiency (i.e., did not achieve more output with less spending) [75].
- Leads to avoidable deaths. Persistent use of cost containment in healthcare has been linked to an increase in avoidable deaths [73,76]. A UK analysis of healthcare spending cuts between 2010 and 2015 showed that a 13.6% reduction in healthcare spending was associated with an additional 33,888 deaths [76]. In Italy, spending cuts implemented from 2007 to 2010 were associated with a 3% increase in avoidable deaths between 2007 and 2014 [77].
- Delays access to innovation (treatments). Time to market of new medicines is strongly inversely correlated to the size of a country’s healthcare budget per capita [4]. In countries with health expenditure above the EU27 average, more new medicines are made available to patients more quickly [78]. An analysis also found that a 10% drop in the price of medicines in the EU often led to an 8% increase in the delay of access to medicines [79].
- Discourages R&D of new medicines. Research shows that lifting pharmaceutical price regulations in 32 OECD countries (including 27 European countries) in 2018 would have increased the global pharmaceutical sales revenue by USD 254 billion, resulting in USD 56 billion in additional R&D expenditures and the development of 25 new drugs annually [42].
- Jeopardises competitiveness. A pharmaceutical competitiveness gap is emerging in the EU due to lower spending on innovation, longer regulatory timelines, and greater access challenges, as recently signposted in the Future of European Competitiveness Report by Mario Draghi [4]. Evidence shows that the EU is falling behind in most dynamic market segments. For example, only two of the top ten best-selling biological medicines in Europe in 2022 were marketed by EU companies, compared to six (including the top four) by U.S.-based companies, and none of the best-selling orphan medicines in the EU/EEA in 2022 were marketed by EU-based companies [4]. In the last two decades, Europe has seen a decline in its global share of pharmaceutical R&D investment, clinical trials, and manufacturing output. The situation is most acute for Advanced Therapies Medicinal Products (ATMPs)—tissue, gene and cell therapies—used to prevent, treat and cure rare conditions, including some cancers [80].
- Results in negative economic impact. Cost containment in healthcare can cause negative economic spillovers, loss of jobs and productivity. A recent analysis estimates that retaining high rates of clawbacks in the UK may result in a loss of over £50 billion in economic output, foregoing a further £90 billion of GDP and about £30 billion in associated tax revenues [81].

Policymakers must learn from the impact of previous cost-containment policies to avert future detriments to population health and economic recovery.

## 6. Delivering Optimal Healthcare Under Constrained Budgets

We recognise that Europe, like other regions, faces a dual challenge: rising health demands from ageing populations and chronic diseases, and a challenging fiscal environment marked by slow economic growth. Rising healthcare costs also present a unique challenge. Therefore, it is not as simple as continuously increasing investment and spending; Europe must make smarter investments with the limited resources available to ensure system sustainability.

We maintain that cost containment is not an effective solution. Cost containment is driven by short-term gains at the expense of long-term consequences. Pressure to cut costs means that health expenditure is no longer aligned with growing healthcare needs and constrains economic performance [74]. There is a more constructive way to deliver optimal healthcare under tight budgets: by shifting focus to a long-term view, eliminating waste, moving toward value-driven approaches, and realigning incentives. Policymakers should consider the following actions.

- Governments must adopt a long-term vision, move away from short-term thinking and value health appropriately to drive economic growth. The principle of discounting in economics reflects a preference for immediate financial returns over future gains. This short-term focus poses a challenge in policymaking, where short electoral cycles and the need for quick political wins often drive shortsighted decisions, such as those seen lately in healthcare. The evidence consistently demonstrates that long-term health and socioeconomic losses often exceed short-term savings achieved through cost containment. Policymakers should prioritise increasing strategic investments in healthcare to safeguard population health now and in the future. However, we acknowledge that there is wide variation across European countries in terms of their investment in new and innovative medicines, suggesting that some countries may wish to reconsider how their lower investment as a share of GDP impacts their patient, health and societal outcomes [82].
- Transformative policies that eliminate ineffective and wasteful spending can generate greater efficiency gains than traditional cost-control approaches. The OECD estimates that eliminating half of ineffective and wasteful health spending could yield savings compared to otherwise projected spend, equivalent to around 1.2 percentage points of GDP, placing health systems on a more moderated expenditure path, with total health spending reaching about 10.6% of GDP by 2040 under a transformative policy scenario (and 11.8% of GDP without such reforms) [74]. Such transformative policies include reducing medical errors and inappropriate antimicrobial use, fostering better use of health data, and improving the management of critical care resources. Savings generated from eliminating waste would create headroom to increase spending on value-based strategies and innovation.
- Value-based strategies are key to sustainably manage healthcare spend, improve access, and system efficiency. HTA frameworks must keep pace with scientific innovation and evolve to capture the breadth of value that new technologies bring to patients, carers, and society in a deliberative manner. Connecting spending on innovative technologies with the value they deliver could not only avoid wasteful and ineffective resource use but also help manage affordability and incentivise innovation. Value-based strategies include: (i) shifting perspective to reward the delivery of health outcomes and economic benefits; (ii) redefining value assessment to adopt a holistic view, capturing what matters most to patients and society; and (iii) optimising the integration of new technologies within the care pathway to benefit patient experience and system sustainability. We believe the EU HTA regulation has the potential to streamline processes and enable faster access decisions at the Member State level by harmonising clinical assessment across Europe; however, more needs to be done to ensure that value assessment frameworks capture the holistic benefit of innovative technologies, and to prevent local HTA from being misused as a cost-cutting measure.
- Incentives and rewards for innovation that address high unmet needs are critical. Research shows that current innovations could reduce the burden of poor health, yet further investment in new solutions is essential because, by 2040, 50% of future ill health in Europe will not be addressed by current innovations [16]. We must not forget that today’s innovative therapies will become tomorrow’s generics, and sustained investment in innovation is crucial to ensure their continued availability. Indeed, this product lifecycle provides the headroom for investment in future innovation to enable continued improvements in health outcomes.
- European countries must therefore strategically incentivise pharmaceutical R&D to meet the projected health needs. To do this, European and national policymakers need to prioritise and scale targeted investments to support R&D infrastructure, especially in expanding digital capabilities, such as artificial intelligence (AI), to allow for new innovations (in preventive and curative care, as well as in areas with high unmet needs) to be developed and ensure a stable, attractive and predictable Regulatory and IP framework [83,84,85]. Policymakers also need to maintain attractive markets by rewarding the value of innovations, thereby linking a country’s health investment to outcomes [86].

These actions are urgently needed in Europe. The cost of inaction is stark. As research shows, if we continue with the same levels of health investment relative to GDP, rather than increasing it, life expectancy at age 65 could decrease by four years starting in 2050 [87].

There are some flagship European initiatives, such as the EU Life Sciences Strategy and the forthcoming Biotech Act, that signal political interest in addressing some of the issues we have highlighted. We welcome these initiatives, which provide a unifying framework for aligning research, innovation, and industrial policy objectives to strengthen Europe’s biotech ecosystem and accelerate the translation of scientific advances into patient benefit. However, policymakers at the European and national levels must ensure strong alignment and coherence so that national policies are not at variance with the objectives of these European initiatives [88].

## 7. Conclusions

Healthcare policy in Europe is at a critical inflection point. The future of healthcare in Europe is reliant on stronger and sustained investment [11,12]. If the European Commission is to succeed in its strategy for European Life Sciences and position Europe as the world’s most attractive place for life sciences by 2030, there needs to be a fundamental shift to eliminate certain forms of cost containment.

We know that there is widespread waste which, if targeted, could not only improve system efficiencies but also create headroom for investment in health innovations. We also know from the evidence presented that the return on investment in health and pharmaceutical innovations is positive, not only bringing direct health benefits to patients but also contributing to population prosperity and economic growth.

We therefore call on regional and national policymakers in Europe to:

- Shift mindset away from short-term and siloed thinking and allow health budgets to rise relative to needs, recognising that returns may not be realised within the same sector, and may be long-term.
- Create headroom for innovation by implementing the transformative policies highlighted in this paper to eliminate waste. These would lead to greater efficiencies and improve affordability more than cost-control policies.
- Focus on value by adopting value-based approaches to link spending to outcomes, thereby maximising value for patients and society.
- Implement fiscal policies that recognises and rewards innovation by creating incentives that attract greater investments into Europe’s innovation ecosystem to strengthen the region’s competitiveness and safeguard Europe’s future.

## Data Availability

No new data were created or analyzed in this study.

## References

[1] van Lerven F., Caddick D., Mang S., Suttor-Sorel L. (2022). Europe’s Fiscal Framework—The People’s View?. https://neweconomics.org/uploads/files/NEF_Europes-Fiscal-Framework.pdf.

[2] Stuckler D., Reeves A., Loopstra R., Karanikolos M., McKee M. (2017). Austerity and Health: The Impact in the UK and Europe. Eur. J. Public Health.

[3] European Commission (2025). Reform of the EU Pharmaceutical Legislation. https://health.ec.europa.eu/medicinal-products/legal-framework-governing-medicinal-products-human-use-eu/reform-eu-pharmaceutical-legislation_en.

[4] Draghi M. (2024). The Future of European Competitiveness. https://commission.europa.eu/topics/eu-competitiveness/draghi-report_en.

[5] European Commission Social Europe—April 2024—Eurobarometer Survey. https://europa.eu/eurobarometer/api/deliverable/download/file?deliverableId=92238.

[6] Eurostat (2025). Preventable and Treatable Mortality Statistics. https://ec.europa.eu/eurostat/statistics-explained/index.php?title=Preventable_and_treatable_mortality_statistics.

[7] Sheridan P.E., Mair C.A., Quiñones A.R. (2019). Associations between prevalent multimorbidity combinations and prospective disability and self-rated health among older adults in Europe. BMC Geriatr..

[8] Bray F., Laversanne M., Sung H., Ferlay J., Siegel R.L., Soerjomataram I., Jemal A. (2024). Global Cancer Statistics 2022: GLOBOCAN Estimates of Incidence and Mortality Worldwide for 36 Cancers in 185 Countries. CA A Cancer J. Clin..

[9] European Commission Mental Health—October 2023—Eurobarometer Survey. https://europa.eu/eurobarometer/api/deliverable/download/file?deliverableId=88914.

[10] World Health Organization European Health Report 2024: Keeping Health High on the Agenda. https://iris.who.int/bitstream/handle/10665/380381/9789289061704-eng.pdf?sequence=2.

[11] OECD Health at a Glance: Europe 2024: State of Health in the EU Cycle. https://www.oecd.org/en/publications/health-at-a-glance-europe-2024_b3704e14-en.html.

[12] OECD (2025). Health at a Glance 2025: OECD Indicators. https://www.oecd.org/en/publications/health-at-a-glance-2025_8f9e3f98-en.html.

[13] IQVIA (2025). Drug Expenditure Dynamics 2000–2022. https://www.iqvia.com/insights/the-iqvia-institute/reports-and-publications/reports/drug-expenditure-dynamics-2000-2022.

[14] IQVIA (2022). Understanding Net Pharmaceutical Expenditure Dynamics in Europe. https://www.iqvia.com/-/media/iqvia/pdfs/library/white-papers/iqvia-understanding-net-pharmaceutical-expenditure-dynamics-in-europe.pdf.

[15] EFPIA and Vintura (2023). Broadening the Perspective: Recommendations for Improving Pharmaceutical Affordability. https://www.efpia.eu/media/v04jfgjb/recommendations-for-improving-pharmaceutical-affordability.pdf.

[16] McKinsey Global Institute (2021). How Keeping Health a Priority Is a Prescription for European Prosperity. https://www.mckinsey.com/industries/healthcare/our-insights/how-keeping-health-a-priority-is-a-prescription-for-european-prosperity.

[17] Bloom D.E., Canning D., Kotschy R., Prettner K., Schünemann J. (2024). Health and Economic Growth: Reconciling the Micro and Macro Evidence. World Dev..

[18] Scott A., Ashwin J., Ellison M., Sinclair D. (2023). International Gains to Achieving Healthy Longevity. Cold Spring Harb. Perspect. Med..

[19] Hofmarcher T., Lindgren P., Wilking N., Jönsson B. (2020). The Cost of Cancer in Europe 2018. Eur. J. Cancer.

[20] Uyl-de Groot C.A., Heine R., Krol M., Verweij J. (2020). Unequal Access to Newly Registered Cancer Drugs Leads to Potential Loss of Life-Years in Europe. Cancers.

[21] OECD, OECD/European Union (2018). Health at a Glance: Europe 2018: State of Health in the EU Cycle. https://www.oecd.org/content/dam/oecd/en/publications/reports/2018/11/health-at-a-glance-europe-2018_g1g91fe4/health_glance_eur-2018-en.pdf.

[22] Arias D., Saxena S., Verguet S. (2022). Quantifying the Global Burden of Mental Disorders and Their Economic Value. eClinicalMedicine.

[23] Masters R., Anwar E., Collins B., Cookson R., Capewell S. (2017). Return on Investment of Public Health Interventions: A Systematic Review. J. Epidemiol. Community Health.

[24] Stelmach R., Kocher E.L., Kataria I., Jackson-Morris A.M., Saxena S., Nugent R. (2022). The Global Return on Investment from Preventing and Treating Adolescent Mental Disorders and Suicide: A Modelling Study. BMJ Glob. Health.

[25] Bell J., Berry T., Deanfield J., Hassan I., Joshi R., Schindler Y., Scott A. (2024). Prosperity Through Health: The Macroeconomic Case for Investing in Preventative Health Care in the UK. https://institute.global/insights/economic-prosperity/the-macroeconomic-case-for-investing-in-preventative-health-care-UK.

[26] World Health Organization (2019). Immunization. https://www.who.int/news-room/facts-in-pictures/detail/immunization.

[27] Banhawi H., Chowdhury S., Neri M., Radu P., Hodgson S., Bell E., Brassel S. (2024). Steuten Socio-Economic Value of Adult Immunisation Programmes—OHE. https://www.ohe.org/publications/the-socio-economic-value-of-adult-immunisation-programmes/socio-economic-value-of-adult-immunisation/.

[28] Zhou F., Jatlaoui T.C., Leidner A.J., Carter R.J., Dong X., Santoli J.M., Stokley S., Daskalakis D.C., Peacock G. (2024). Health and Economic Benefits of Routine Childhood Immunizations in the Era of the Vaccines for Children Program—United States, 1994–2023. MMWR Morb. Mortal. Wkly. Rep..

[29] Bencina G., Sabale U., Morais E., Ovcinnikova O., Oliver E., Shoel H., Meiwald A., Hughes R., Weston G., Sundström K. (2024). Burden and Indirect Cost of Vaccine-Preventable Cancer Mortality in Europe. J. Med. Econ..

[30] World Health Organization (2025). Promoting Cancer Early Diagnosis. https://www.who.int/activities/promoting-cancer-early-diagnosis.

[31] Duffy S., Vulkan D., Cuckle H., Parmar D., Sheikh S., Smith R., Evans A., Blyuss O., Johns L., Ellis I. (2020). Annual Mammographic Screening to Reduce Breast Cancer Mortality in Women from Age 40 Years: Long-Term Follow-up of the UK Age RCT. Health Technol. Assess..

[32] Diaz M., Garcia M., Vidal C., Santiago A., Gnutti G., Gómez D., Trapero-Bertran M., Fu M. (2021). Health and Economic Impact at a Population Level of Both Primary and Secondary Preventive Lung Cancer Interventions: A Model-Based Cost-Effectiveness Analysis. Lung Cancer.

[33] OECD (2024). Beating Cancer Inequalities in the EU: Spotlight on Cancer Prevention and Early Detection. https://www.oecd.org/en/publications/beating-cancer-inequalities-in-the-eu_14fdc89a-en.html.

[34] World Health Organization (2017). Early Cancer Diagnosis Saves Lives, Cuts Treatment Costs. https://www.who.int/news/item/03-02-2017-early-cancer-diagnosis-saves-lives-cuts-treatment-costs.

[35] Gill J., Fontrier A.-M., Miracolo A., Kanavos P. (2020). Access to Personalised Oncology in Europe. https://www.lse.ac.uk/business/consulting/assets/documents/Personalised-Oncology-in-Europe.pdf.

[36] Murphy C., Byrne S., Ahmed G., Kenny A., Gallagher J., Harvey H., O’Farrell E., Bird B. (2018). Cost Implications of Reactive Versus Prospective Testing for Dihydropyrimidine Dehydrogenase Deficiency in Patients with Colorectal Cancer: A Single-Institution Experience. Dose Response.

[37] Lichtenberg F.R. (2022). The Effect of Pharmaceutical Innovation on Longevity: Evidence from the U.S. and 26 High-Income Countries. Econ. Hum. Biol..

[38] Angelis R.D., Demuru E., Baili P., Troussard X., Katalinic A., Lopez M.D.C., Innos K., Santaquilani M., Blum M., Ventura L. (2024). Complete Cancer Prevalence in Europe in 2020 by Disease Duration and Country (EUROCARE-6): A Population-Based Study. Lancet Oncol..

[39] EFPIA (2025). Cancer Survival. https://www.efpia.eu/publications/cancer-comparator-report-2025/survival/.

[40] Trickey A., Sabin C.A., Burkholder G., Crane H., d’Arminio Monforte A., Egger M., Gill M.J., Grabar S., Guest J.L., Jarrin I. (2023). Life Expectancy after 2015 of Adults with HIV on Long-Term Antiretroviral Therapy in Europe and North America: A Collaborative Analysis of Cohort Studies. Lancet HIV.

[41] PhRMA (2017). Prescription Medicines: International Costs in Context. https://phrma.org/resources/prescription-medicines-international-costs-in-context.

[42] Long T., Ezell S. (2023). The Hidden Toll of Drug Price Controls: Fewer New Treatments and Higher Medical Costs for the World. https://itif.org/publications/2023/07/17/hidden-toll-of-drug-price-controls-fewer-new-treatments-higher-medical-costs-for-world/.

[43] Chaplin S. (2020). Monoclonal Antibodies for the Treatment of Severe Asthma. Prescriber.

[44] Zozaya N., Alcalá B., Galindo J. (2019). The Offset Effect of Pharmaceutical Innovation: A Review Study. Glob. Reg. Health Technol. Assess..

[45] Costello R.W., Long D.A., Gaine S., Mc Donnell T., Gilmartin J.J., Lane S.J. (2011). Therapy with Omalizumab for Patients with Severe Allergic Asthma Improves Asthma Control and Reduces Overall Healthcare Costs. Ir. J. Med. Sci..

[46] EFPIA (2022). Demonstrating the Value of Medical Innovation in Europe. https://www.efpia.eu/media/676701/efpia-power-of-innovation.pdf.

[47] Bui V., Stolpe M. (2010). The Impact of New Drug Launches on the Loss of Labor from Disease and Injury: Evidence from German Panel Data. Int. J. Health Care Financ. Econ..

[48] Stadhouders N., Koolman X., Tanke M., Maarse H., Jeurissen P. (2016). Policy Options to Contain Healthcare Costs: A Review and Classification. Health Policy.

[49] Donato A.A., Pita J.R., Batel-Marques F. (2022). Classification of Pharmaceutical Policy Measures During the Portuguese Financial Crisis. Inquiry.

[50] Nguyen T.A., Knight R., Roughead E.E., Brooks G., Mant A. (2015). Policy Options for Pharmaceutical Pricing and Purchasing: Issues for Low- and Middle-Income Countries. Health Policy Plan..

[51] Carone G., Schwierz C., Xavier A. (2012). Cost-Containment Policies in Public Pharmaceutical Spending in the EU. https://ssrn.com/abstract=2161803.

[52] NHS (2021). Commercial Framework for New Medicines. https://www.england.nhs.uk/long-read/nhs-commercial-framework-for-new-medicines/.

[53] Vogler S., Zimmermann N., Leopold C., de Joncheere K. (2011). Pharmaceutical Policies in European Countries in Response to the Global Financial Crisis. South Med. Rev..

[54] Leopold C., Mantel-Teeuwisse A.K., Vogler S., Valkova S., de Joncheere K., Leufkens H.G., Wagner A.K., Ross-Degnan D., Laing R. (2014). Effect of the Economic Recession on Pharmaceutical Policy and Medicine Sales in Eight European Countries. Bull. World Health Organ.

[55] Mladovsky P., Srivastava D., Cylus J., Karanikolos M., Evetovits T., Thomson S., McKee M. (2012). Health Policy Responses to the Financial Crisis in Europe. https://iris.who.int/handle/10665/108608.

[56] Howey W. (2023). EU Pharma Overhaul Favours Access over Innovation. https://www.eiu.com/n/eu-pharma-overhaul-favours-access-over-innovation/.

[57] (2024). Smart Pharma Healthcare Costs Regulation in France. https://smart-pharma.com/wp-content/uploads/2025/01/Healthcare-Costs-Regulation-in-France-VF.pdf.

[58] Koyuncu D.D.A. (2022). Germany Significantly Tightens Drug Pricing and Reimbursement Laws. https://www.insideeulifesciences.com/2022/10/26/germany-significantly-tightens-drug-pricing-and-reimbursement-laws/.

[59] (2025). Denmark Announces Agreement to Reduce Hospital Medicine Prices by 5% Over Three Years. https://www.navlindaily.com/article/24375/denmark-announces-agreement-to-reduce-hospital-medicine-prices-by-5-over-three-years.

[60] Economic Committee for Health Products (2023). Notice Relating to the Prices of Pharmaceutical Specialties. https://www.legifrance.gouv.fr/jorf/id/JORFTEXT000048572544#.

[61] Vital Transformation (2024). The EU General Pharmaceutical Legislation & Clawbacks: Calculated Impacts—Both Designed and Unintended. https://vitaltransformation.com/wp-content/uploads/2024/04/EU_GPL_Clawbacks_Final_4.18.24-2.pdf.

[62] Letsios A.N., Mavridoglou G., Ladopoulou D., Tsourdini D., Dedes N., Polyzos N.M. (2023). Exploring the Impact of Clawback on Pharmaceutical Expenditure: A Case Study of Public Hospitals in Greece. Int. J. Health Plan. Manag..

[63] Cohen J.P. (2023). U.K.’s Voluntary Scheme for Branded Medicines, Pricing, And Access (VPAS) Faces A Potential Crisis. https://www.forbes.com/sites/joshuacohen/2023/01/19/uks-voluntary-scheme-for-branded-medicines-pricing-and-access-vpas-faces-a-potential-crisis/.

[64] Schaefer R., Hernández D., Bärnighausen T., Kolominsky-Rabas P., Schlander M. (2023). Health Technology Assessment–Informed Decision Making by the Federal Joint Committee/Institute for Quality and Efficiency in Health Care in Germany and the National Institute for Health and Care Excellence in England: The Role of Budget Impact. Value Health.

[65] Centre for Innovation in Regulatory Science Review of HTA Outcomes and Timelines in Australia, Canada, Europe and the UK 2019–2023. https://www.cirsci.org/publications/cirs-rd-briefing-95-review-of-hta-outcomes-and-timelines-in-australia-canada-europe-and-the-uk-2019-2023/.

[66] APM Health Europe IQVIA—Over Half of Requests for Pre-Approval Early-Stage Access Schemes Refused in France in 2024. https://www.apmmarketaccess.com/story.php?objet=92084.

[67] Watt A. Risk-Sharing Agreements Are Growing at a Rate of 24%. https://www.pharmaceutical-technology.com/pricing-and-market-access/risk-sharing-agreements/.

[68] European Commission Implementation of the Regulation on Health Technology Assessment—Public Health. https://health.ec.europa.eu/health-technology-assessment/implementation-regulation-health-technology-assessment_en.

[69] Danish Medicines Council (2023). Medicinrådet Nedlægger Ansøgningsproces for Biosimilære Lægemidler. https://medicinraadet.dk/nyheder/2023/medicinradet-nedlaegger-ansogningsproces-for-biosimilaere-laegemidler.

[70] Jeremias S. (2024). Review Highlights Most Popular European Policies to Boost Biosimilar Uptake. https://www.centerforbiosimilars.com/view/review-highlights-most-popular-european-policies-to-boost-biosimilar-uptake.

[71] Doetsch J.N., Schlösser C., Barros H., Shaw D., Krafft T., Pilot E. (2023). A Scoping Review on the Impact of Austerity on Healthcare Access in the European Union: Rethinking Austerity for the Most Vulnerable. Int. J. Equity Health.

[72] Michalopoulos S. OECD Official: 20% of All Health Spending in Europe Is Pure Waste. https://www.euractiv.com/section/health-consumers/interview/oecd-official-20-of-all-health-spending-in-europe-is-pure-waste/.

[73] Guccio C., Pignataro G., Romeo D., Vidoli F. (2024). Is Austerity Good for Efficiency, at Least? A Counterfactual Assessment for the Italian NHS. Socio-Econ. Plan. Sci..

[74] OECD (2024). Fiscal Sustainability of Health Systems: How to Finance More Resilient Health Systems When Money Is Tight?. https://www.oecd.org/content/dam/oecd/en/publications/reports/2024/01/fiscal-sustainability-of-health-systems_c2f837ac/880f3195-en.pdf.

[75] Giancotti M., Sülkü S., Pipitone V., Mauro M. (2020). Do Recovery Plans Improve Public Hospitals Efficiency and Productivity? Evidence from Italy. Int. Rev. Bus. Res. Pap..

[76] Martin S., Longo F., Lomas J., Claxton K. (2021). Causal Impact of Social Care, Public Health and Healthcare Expenditure on Mortality in England: Cross-Sectional Evidence for 2013/2014. BMJ Open.

[77] Arcà E., Principe F., Van Doorslaer E. (2020). Death by Austerity? The Impact of Cost Containment on Avoidable Mortality in Italy. Health Econ..

[78] EFPIA (2025). New Data Shows No Shift in Access to Medicines for Millions of Europeans. https://www.efpia.eu/news-events/the-efpia-view/statements-press-releases/new-data-shows-no-shift-in-access-to-medicines-for-millions-of-europeans/.

[79] Schulthess D., Bowen H. (2021). The Historical Impact of Price Controls on the Biopharma Industry. https://vitaltransformation.com/2021/11/the-historical-impact-of-price-controls-on-the-biopharma-industry/.

[80] Wilsdon T., Armstrong H., Sablek A., Cheng P. (2022). Factors Affecting the Location of Biopharmaceutical Investments and Implications for European Policy Priorities. https://www.efpia.eu/media/676753/cra-efpia-investment-location-final-report.pdf.

[81] Hughes S. (2023). False Economy: How NHS Medicine Procurement Threatens the UK’s Life Sciences Growth Engine. https://www.abpi.org.uk/media/1ebnqexr/false-economy-additional-analysis-231009.pdf.

[82] PhRMA (2025). High-Income Country Spending on Innovative Medicines. https://www.phrma.org/resources/high-income-country-spending-on-innovative-medicines.

[83] European Observatory on Health Systems and Policies (2023). Financing for Health System Transformation: Spending More or Spending Better (or Both)?. https://eurohealthobservatory.who.int/publications/i/financing-for-health-system-transformation-spending-more-or-spending-better-(or-both).

[84] Deloitte Insights (2024). The Future of Health in Europe. https://www.deloitte.com/content/dam/assets-zone2/ch/en/docs/industries/life-sciences-health-care/2024/deloitte-ch-lshc-the-future-of-health-in-europe.pdf.

[85] United States Government Accountability Office (2019). Artificial Intelligence in Health Care: Benefits and Challenges of Machine Learning in Drug Development. https://www.gao.gov/assets/gao-20-215sp.pdf.

[86] EFPIA (2024). A Competitiveness Strategy for European Life Sciences. https://efpia.eu/media/fzkbhzoe/a-competitiveness-strategy-for-european-life-sciences.pdf.

[87] Böhm S., Grossmann V., Strulik H. (2021). R&D-driven medical progress, health care costs, and the future of human longevity. J. Econ. Ageing.

[88] European Policy Centre (2025). Europe’s Competitiveness Challenge: Ensuring Patient Access to Transformative Therapies in Europe. https://www.epc.eu/publication/europes-competitiveness-challenge-ensuring-patient-access-to-transformative-therapies-in-europe/.

